# Supplementary material for: Dynalign II: common secondary structure prediction for RNA homologs with domain insertions
Source: Nucleic Acids Res. 2014 Nov 21;42(22):13939–48. doi: 10.1093/nar/gku1172 (PMC4267632; doi:10.1093/nar/gku1172)
Supplement: SUPPLEMENTARY DATA [file supp_gku1172_nar-02021-z-2014-File012.zip › manual/Text/draw.html]

RNAstructure Command Line Help: draw


|  |  |  |
| --- | --- | --- |
|  | RNAstructure Command Line Help draw | - Contents - Index |
| draw is used to draw secondary structures as Postscript images. These Postscript images can be drawn in a variety of ways, including annotated or circularized. Every individual structure is sized to fit on a single Postscript page. USAGE: draw <ct file> <ps file> [options] Required parameters: <ct file> \| The name of a CT file containing data for the structure to be drawn.; &ltoutput file> \| The name of an image file to which output will be written. Usually, this is a Postscript image file, although the user can specify that it be an SVG image file instead. To specify SVG images, the \"--svg\" flag must be specified in conjunction with a structure number flag (see the flags below). Options which don't require added values: -c, -C, --circle \| Specify that the structure should be drawn with its backbone around a circle. Note that pseudoknotted structures will be drawn circularized by default even if this option is not specified. Default is to show a collapsed structure.; -f, -F, --flat \| Specify that the structure should be drawn with its backbone stretched in a straight line. Note that pseudoknotted structures can be drawn linearized if this option is specified. Default is to show a collapsed structure.; -h, -H, --help \| Display the usage details message.; -l, -L, --levorotatory \| Specify that the drawn structure is rendered counterclockwise. Default is to render drawn structures clockwise.; --svg \| Specify that the output file should be an SVG image file, rather than a Postscript image file. Note that only one SVG image can be written into a particular file, so the structure number flag (below) must also be specified when writing an SVG document.; -u, -U, --uncircled \| Specify that no circles should surround nucleotides when drawing. Default is to surround nucleotides with circles. Options which require added values: -n, -N, --number \| Specify a particular structure to be output, one-indexed. Default is -1, which signifies all structures output to one file.; -p, -P, --probability \| Specify the name of the file from which base pairing probability data will be read for annotation. This file must be a partition function save file generated by the partition interface. Default is no probability annotation file used.; -s, -S, --SHAPE \| Specify the name of the SHAPE annotation file from which SHAPE data will be read for annotation. Default is no SHAPE annotation file used.; -t, -T, --text \| Specify the name of the text file from which base pairing probability data will be read for annotation. This file should describe pairing data for the predicted structure, not the accepted structure. Default is no probability annotation file used. References: 1. Reuter, J.S. and Mathews, D.H. "RNAstructure: software for RNA secondary structure prediction and analysis." *BMC Bioinformatics*, 11:129. (2010). | | |
| Visit The Mathews Lab RNAstructure Page for updates and latest information. | | |
